# The Components of *Drosophila* Histone Chaperone dCAF-1 Are Required for the Cell Death Phenotype Associated with *rbf1* Mutation

**DOI:** 10.1534/g3.113.007419

**Published:** 2013-10-01

**Authors:** Heather Collins, Nam-Sung Moon

**Affiliations:** Department of Biology, Developmental Biology Research Initiative, McGill University, Montreal, Quebec H3A 1B1, Canada

**Keywords:** *rbf1*, posterior sex combs, chromatin assembly factor-1, cell death

## Abstract

A Polycomb group protein, Posterior sex combs (Psc), was identified in a genetic screen designed to find factors that can specifically induce morphological defects in *rbf1* mutant eyes. We discovered that *rbf1* mutations enhance developmental phenotypes caused by Psc overexpression such as ectopic cell death and disorganized ommatidia. Our genetic analysis revealed that Psc-induced developmental defects are strongly influenced by CAF1p55, which is a shared component of several chromatin-associated complexes including a histone chaperone complex, chromatin assembly factor-1 (dCAF-1). Interestingly, the expression levels of dCAF-1 components, CAF1p105 and CAF1p180, are increased in *rbf1* mutants, whereas the expression level of CAF1p55 itself remains relatively unchanged. We demonstrated that the increased levels of CAF1p105 and CAF1p180 are required for the hypersensitivity of *rbf1* mutant cells to Psc-induced cell death and for the developmentally regulated cell death normally observed in *rbf1* mutant eyes. We propose that *Caf1p105* and *Caf1p180* are important determinants of cell death sensitivity in *rbf1* mutant cells and contribute to the genetic interaction between *Psc* and *rbf1*.

Retinoblastoma protein (pRB) is a tumor suppressor protein that was first discovered as a result of its deletion in rare childhood retinal cancers called retinoblastomas (Friend *et al.* 1986). pRB is conserved across metazoans and regulates a number of important developmental processes, including proliferation, apoptosis, and cell-type specification (van den Heuvel and Dyson 2008). The role of pRB in cell-cycle regulation is well-studied and occurs through its interaction with E2F transcription factors in G1 to regulate the expression of E2F target genes. pRB activity is modulated through the tightly controlled phosphorylation of Ser and Thr residues by cyclin-dependent kinases (CDKs). Hyperphosphorylated pRB is inactive and unable to bind E2F transcription factors. Importantly, the pRB/E2F pathway is altered in the majority of human cancers either by lesions at the pRB locus or by the functional inactivation of pRB via CDK \hyperactivation or CDK inhibitor inactivation. In mice, loss of pRB results in uncontrolled cell proliferation, apoptosis, and changes in cell fate (Calo *et al.* 2010; Clarke *et al.* 1992; Jacks *et al.* 1992; Lee *et al.* 1992; Macleod *et al.* 1996).

Although pRB inactivation is an initiating event in the development of many different types of tumors, cells must accrue additional changes to form aggressive tumors. For example, p53 is another tumor suppressor protein whose activity is commonly inactivated in cancer (Vogelstein *et al.* 2000). Even in retinoblastomas where genetic lesions in the *p53* gene are not found, a negative regulator of *p53*, *MdmX*, is amplified to inactivate the p53 pathway (Kato *et al.* 1996; Laurie *et al.* 2006). Interestingly, a recent study showed that human retinoblastomas contain many epigenetic changes, such as histone modifications and DNA methylation, at the genomic loci of genes involved in multiple signaling pathways (Zhang *et al.* 2012). The same study also demonstrated that the expression of spleen tyrosine kinase (*Syk*) is increased by epigenetic changes in retinoblastomas and is required for the survival of retinoblastoma cells, making *Syk* a potential therapeutic target (Zhang *et al.* 2012). Overall, both genetic and epigenetic changes occur during retinoblastoma development and likely cooperate with pRB mutations to promote cancer progression. Therefore, identifying factors that can cooperate with pRB deficiency may improve our understanding of pRB mutant cancer cells.

Polycomb group (PcG) proteins are epigenetic repressors that maintain gene silencing of important regulators of development, such as Hox genes. PcG proteins form evolutionary-conserved multimeric polycomb repressive complex 1 and polycomb repressive complex 2 (PRC1 and PRC2) that can function together to silence PcG target genes (Simon and Kingston 2009). In *Drosophila melanogaster*, PRC2 contains a methyltransferase subunit, enhancer of zeste (EZ; EZH1 and EZH2 in mammals), which first dimethylates and trimethylates histone H3 lysine 27 (H3K27). The trimethylation of H3K27 is then recognized by PRC1, which is thought to maintain epigenetic silencing by compacting chromatin and inhibiting ATP-dependent nucleosome remodeling. Among the PRC1 components, Psc is shown to inhibit transcription and chromatin remodeling *in vitro*, independently of the other PRC1 components (King *et al.* 2005). Interestingly, BMI-1, the mammalian homolog of Psc, is a known oncogene whose upregulation correlates with poor prognosis in a number of cancers (Guo *et al.* 2011; Qin *et al.* 2009; Song *et al.* 2010). Other PcG genes are also found to be deregulated in cancers, highlighting the importance of the PcG-mediated epigenetic profile during tumorigenesis (Sparmann and van Lohuizenn 2006).

A PRC2 component, CAF1p55, is also part of several other chromatin-associated protein complexes, such as nucleosome remodeling factor, NuRD, dREAM/MMB, and a histone chaperone complex, chromatin assembly factor-1 (dCAF-1). CAF1p55 seems to function as a noncatalytic component of these complexes by promoting chromatin association through binding to histones H3 and H4 (Nowak *et al.* 2011; Song *et al.* 2008). In particular, the dCAF-1 complex, which is composed of CAF1p55, CAF1p180, and CAF1p105, is an important H3/H4 histone chaperone that deposits H3/H4 dimers onto newly synthesized DNA during DNA replication and repair (Eitoku *et al.* 2008). Given the presence of CAF1p55 in multiple chromatin-binding complexes, alteration in CAF1p55 activity may affect histone homeostasis and global gene expression. However, the mechanism by which CAF1p55 activity is regulated is largely unknown.

In *Drosophila melanogaster*, RBF1 is the functional homolog of pRB with the conserved role of negatively regulating the sole transcriptional activator E2F in flies, dE2F1 (Stevaux *et al.* 2002). *Drosophila* provides an excellent genetic system for studying the pRB/E2F pathway in a developmental context. Specifically, eye development in flies can be used to easily screen for defects in cell survival, proliferation, and differentiation. Recently, we identified *extra macrochaetae* (*emc*) from a genetic screen designed to identify factors that can cooperate with *rbf1* mutations (Popova *et al.* 2011). *emc* is the *Drosophila* ortholog of ID family genes in mammals whose function was shown to be important for the developmental defects observed in *pRB* knockout mice (Lasorella *et al.* 2005). We demonstrated that *emc* is also an important differentiation determinant in *rbf1* mutant eyes, validating the use of *Drosophila* as a valuable tool for identifying factors that are important for the pRB/E2F pathway.

In the same genetic screen that identified *emc*, we discovered that *Posterior sex combs* (*Psc*) is a factor that, when overexpressed, can interfere with cell survival and differentiation in *rbf1*-deficient eyes. Further analysis revealed that *rbf1* mutations provide a sensitized genetic background for Psc-induced developmental defects. Our genetic studies revealed that CAF1p55 function is commonly affected by Psc overexpression and *rbf1* mutations. Interestingly, the expression of dCAF-1 components, CAF1p105 and CAF1p180, is upregulated in *rbf1* mutants, whereas the expression level of CAF1p55 remains relatively unchanged. Importantly, we demonstrate that *Caf1p105* and *Caf1p180* are required for the developmentally regulated cell death phenotype in *rbf1* mutant eyes and the Psc-induced cell death phenotype.

## Materials and Methods

### Fly stocks

All crosses were performed at 25°C unless otherwise stated. *rbf1^120a^* is a hypomorphic *rbf1* allele that was previously described (Du and Dyson 1999). f00391 is from the Harvard Exelixis collection and contains a WH insertion element, containing a UAS site, upstream of the *Psc* gene. The overexpression of Psc was performed in wild-type and *rbf1^120a^* background with the following genotypes:

yw ey-FLP/+;f00391/Act5c < CD2 < Gal4 UAS-GFP
rbf1^120a^ ey-FLP/rbf1^120a^;f00391/Act5c < CD2 < Gal4 UAS-GFP

These flies express FLP recombinase under the control of an eye-specific promoter, eyeless (ey), which removes an FRT cassette to allow Gal4 expression from the *Act5c* promoter. f00391 was recombined with GMR-Gal4 to strongly express Psc in the posterior region of the eye disc. To generate *UAS-Psc* flies, the coding sequence of *Psc* was amplified from a cDNA, 1926 pFastBac FPSC, purchased from AddGene, and cloned into pENTR vector. The cassette was recombined into pTFM using LR Clonase and the final construct was injected into *yw* embryos.

*Caf1p55* (stock 105838), *Caf1p105* (stock 110461), and *Caf1p180* (stock 108240) RNAi alleles were obtained from the Vienna Drosophila RNAi Center. The following mutant alleles were obtained from the Bloomington Stock Centre: *E(z)^731^ FRT2a/TM6c*; FRT*82B Sce^1^/TM6C*; *ISWI^KG0335^*; and *mip120^EY0530^/CyO. esc^KG07458^ 40A/CyO* was provided by the Drosophila Genetic Resource Center at the Kyoto Institute of Technology. *FRT82B Caf1^long^, FRT82B Caf1^med^, FRT82B Caf1^short^,* and *UAS-Caf1p55* flies were provided by Dr. Graeme Mardon (Baylor College of Medicine).

### Clone generation

Eye imaginal discs composed largely of homozygous *Caf1p55^long^* cells were generated by mitotic recombination using *ey-FLP and FRT 82B P(W+) l(3)cl-R3*, which carries a recessive lethal gene. For *rbf1* mutant clones, *rbf1^Δ14^* was used to create *rbf1*-null clones.

Clones in the following genotypes were analyzed:

yw ey-FLP/Y;FRT 82B P(W+) l(3)cl-R3/FRT82B Caf1p55^long^
rbf1^120a^ey-FLP/Y;FRT 82B P(W+) l(3)cl-R3/FRT82B Caf1p55^long^
*rbf1^Δ14^*, *FRT19A/GFP^ubi^ FRT19A*; *ey-FLP*

### Immunostaining

For immunostaining, third instar larval and pupal eye imaginal discs were fixed and stained as previously described (Hsieh *et al.* 2010; Popova *et al.* 2011) and visualized using a Zeiss LSM confocal microscope. The following primary antibodies were obtained from Developmental Studies Hybridoma Banks: anti-ELAV (1/100); anti-Psc (1/100); anti-Rough (1/20); and anti-Dlg (1/200). Anti-cleaved caspase-3 was purchased from Cell Signaling Technology (1/100) and Abcam (1/400). Anti-CAF1p180 (1/200) and anti-CAF1p55 (1/400) were purchased from Abcam. Anti-Senseless was a generous gift from Dr. Hugo J. Bellen (Baylor College of Medicine).

### RNA extraction, cDNA synthesis, and quantitative reverse-transcriptase polymerase chain reaction (qRT-PCR)

RNA was extracted from 100 third instar larval eye imaginal discs of each genotype using miRNAeasy Mini Kit (Qiagen). RNA was treated with on-column RNAse-free DNase I digestion (Qiagen) to ensure the elimination of genomic contamination. cDNA was synthesized from 500 ng RNA using the DyNAmo cDNA Synthesis Kit (Finnzymes) with random hexamers. Gene expression was measured in triplicate using DyNAmo Flash SYBR Green qPCR Kit (Finnzymes) and normalized with reference genes *rp49* and *β-tubulin*. ΔΔCt analysis was used to determine the fold change compared with the wild-type and was performed on three independent biological replicates (Livak and Schmittgen 2001). Primers for quantitative reverse-transcriptase were designed using primer3 (Skaletsky 2000) and are as follows:

*rp49* forward TACAGGCCCAAGATCGTGAAG
*rp49* reverse GACGCACTCTGTTGTCGATACC
*β-tubulin* forward ACATCCCGCCCCGTGGTC
*β-tubulin* reverse AGAAAGCCTTGCGCCTGAACATAG
*Caf1p105* forward ACTCGCTTGGTATTGGCATC
*Caf1p105* reverse CAGTGGCAAATCACTGGCTA
*Caf1p180* forward GTCGGCGAAATGCAGATACT
*Caf1p180* reverse AAGGACGAGGAGGATGATGA
*Caf1p55* forward CATCAAAAGGAAGGCTACGG
*Caf1p55* reverse GCCGGTGAAGATGTTCTTG
*Psc* forward CATCAGTTCCCGTTCGTAAAG
*Psc* reverse CGAGATGGTCATCAACAACG

## Results

**Figure 1:**
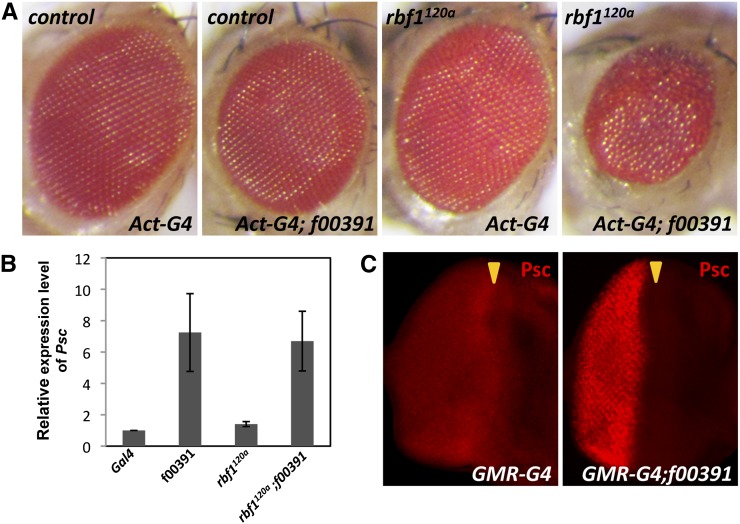
Psc overexpression from Exelixis stock f00391 induces an eye phenotype specifically in an *rbf1* mutant. (A) Adult eyes of control and *rbf1^120a^ Drosophila* display an organized array of ommatidia. This typical pattern of the eye was disrupted by the Exelixis stock f00391 only in *rbf1^120a^* flies. (B) RNA from third instar eye imaginal discs of indicated flies was isolated and the relative levels of *Psc* transcripts were determined qRT-PCR. Levels of *Psc* transcripts from f00391 were the same in control and *rbf1^120a^* eye discs. The average fold change of three independent replicates compared with the control is represented. Error bars indicate the SD of three independent experiments. (C) f00391 was crossed to an eye-specific driver, *GMR-Gal4*, and third instar eye imaginal discs were immunostained with anti-Psc. An eye disc expressing only *GMR-Gal4* also is shown as a control. For all images, the posterior region of the eye disc is to the left, and an arrowhead denotes the position of the morphogenetic furrow.

The Exelixis stock f00391 was identified in a genetic screen designed to identify factors that induce eye phenotypes specifically in sensitized, yet viable, *rbf1^120a^* hypomorphic flies (Popova *et al.* 2011). In brief, Exelixis stocks were used to overexpress random genes using an eye-specific Gal4 driver in control and *rbf1^120a^* flies (described in Materials and Methods). f00391 induced small, disorganized, and rough adult eyes in *rbf1* hypomorphic flies but failed to produce such defects in wild-type flies (Figure 1A). According to the annotated information, this stock contains a WH vector (Thibault *et al.* 2004), which includes a UAS element, inserted upstream of a Polycomb group (PcG) gene, *Psc*. *Psc* transcript levels measured by qRT-PCR confirmed that *Psc* is overexpressed by f00391 and showed that a similar level of *Psc* expression is achieved by f00391 in wild-type and *rbf1^120a^* third instar eye imaginal discs (Figure 1B). Supporting the qRT-PCR data, we also observed an increase in Psc protein levels in the posterior region of eye discs when GMR-Gal4 was used to drive the expression of the gene affected by f00391 (Figure 1C). These results demonstrate that Psc proteins are produced from the Exelixis stock f00391 and that Psc can induce a rough eye phenotype specifically in an *rbf1* mutant.

**Figure 2:**
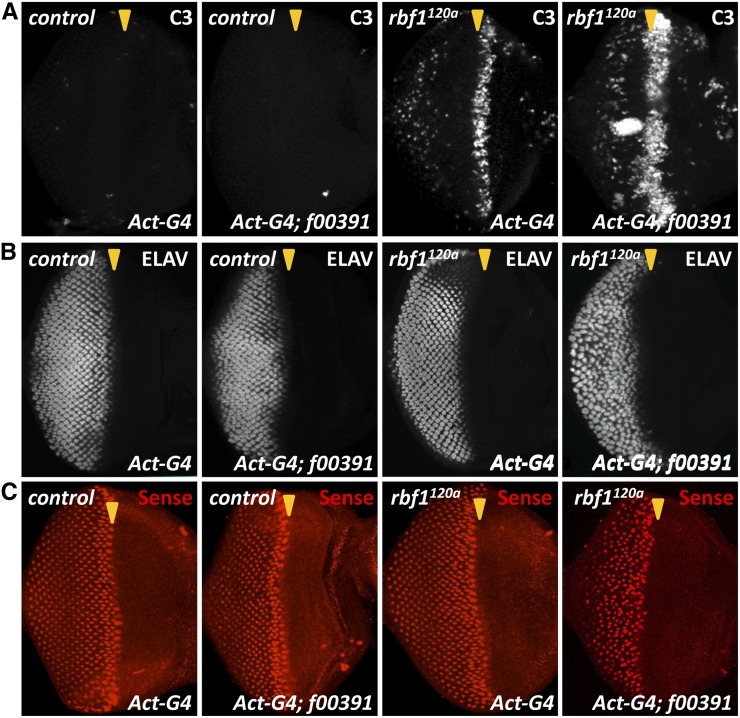
Overexpression of Psc via f00391 causes developmental defects specifically in *rbf1^120a^* hypomorphic eye discs. (A) Third instar eye imaginal discs were stained for cell death using a cleaved caspase-3 antibody (C3). A characteristic stripe of cell death is observed in *rbf1^120a^* eye discs. *rbf1^120a^* eye discs overexpressing Psc showed an increase in ectopic cell death. (B and C) Photoreceptors were visualized by immunostaining for molecular markers, ELAV (all photoreceptors) and Senseless (R8 photoreceptors). ELAV staining showed that Psc overexpression induced disorganization of photoreceptors in *rbf1^120a^* eye discs. Interestingly, Senseless staining showed that although Senseless expression is present at the morphogenetic furrow of *rbf1^120a^* eye discs overexpressing Psc, it failed to remain expressed in the posterior region of the eye discs, suggesting a defect in R8 photoreceptor fate maintenance.

To better characterize the effect of f00391 in *rbf1^120a^* eyes, we searched for specific developmental defects in larval eye imaginal discs using molecular markers for differentiation and cell death. Immunostaining for cleaved caspase-3 (C3) showed that the characteristic stripe of cell death in *rbf1^120a^* eye discs at the morphogenetic furrow was enhanced by f00391 but resulted in no substantial change in a wild-type background (Moon *et al.* 2006; Figure 2A). In addition to the cell death phenotype, f00391 induced defects in ommatidial organization in *rbf1^120a^* eye discs. Whereas the ordered latticework of ommatidia was maintained in control eye discs, f00391 caused a disorganized pattern of ommatidial clusters in *rbf1^120a^* eye discs (Figure 2B). We also found that the expression pattern of Senseless, which is a marker for the R8 photoreceptor, is affected by f00391 in *rbf1^120a^* mutant eye discs (Figure 2C). Although Senseless was expressed at the morphogenetic furrow, its expression was lost in the posterior region of the eye disc. The defect in R8 photoreceptors is likely not the result of increased cell death because we did not detect an overlap between C3 and Senseless stainings (data not shown).

**Figure 3:**
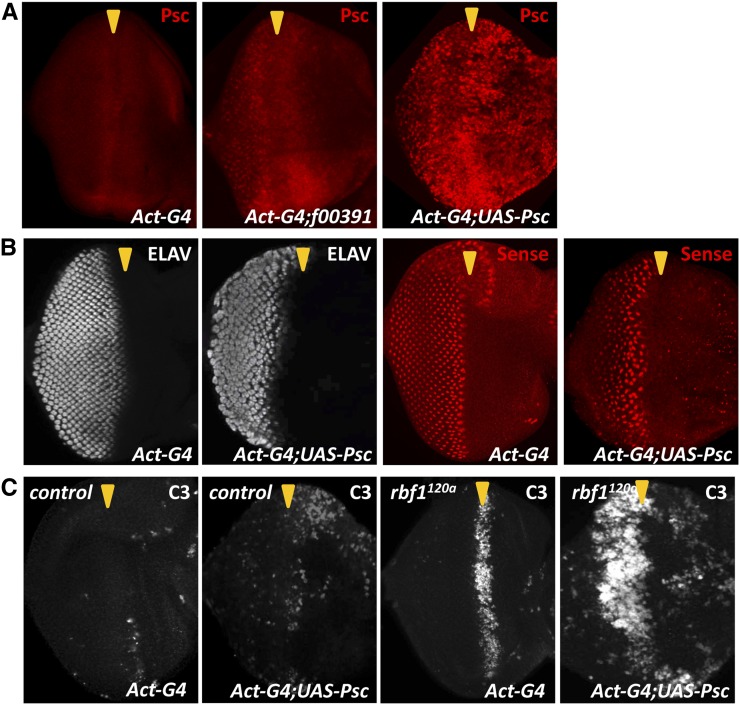
Hyperactivation of Psc is sufficient to recapitulate the phenotypes induced by f00391 in the *rbf1^120a^* mutant background. (A) Psc is overexpressed in wild-type eyes with the same Gal4 driver used in the genetic screen (Act-G4) in combination with either f00391 or a *UAS-Psc* transgene. Note that *UAS-Psc* can achieve a higher level of Psc expression compared with f00391. (B) Third instar eye discs expressing Psc using the UAS-Psc transgene were stained for ELAV and Senseless. Note that high levels of Psc expression induce similar differentiation defects as those observed with f00391 in *rbf1^120a^* eye discs even in a wild-type background. (C) The UAS-Psc transgene was used to express Psc in wild-type and *rbf1^120a^* mutant eye discs and apoptotic cells were visualized using cleaved caspase-3 (C3) antibodies. Note that the UAS-Psc transgene induces ectopic cell death in wild-type eye discs but does not reach the level of cell death observed in *rbf1^120a^* mutant eye discs.

Occasionally, we detected that f00391 was able to induce the Senseless expression defect even in wild-type eye discs (data not shown). This observation led us to hypothesize that *rbf1* mutations may simply enhance the phenotypes associated with Psc overexpression. If this hypothesis is correct, then one would predict that further activating Psc, even in a wild-type background, should recapitulate the phenotypes observed in *rbf1^120a^;f00391* eye discs. To test this idea, we generated a *UAS-Psc* transgene that can express Psc at a much higher level than that achieved by f00391 using the same eye-specific Gal4 driver (Figure 3A). Even in a wild-type background, the *UAS-Psc* construct was able to induce similar developmental defects to those observed in *rbf1^120a^* background by f00391 (Figure 3B). ELAV and Senseless expression patterns demonstrate that the hyperactivation of Psc leads to the same differentiation defects observed in the *rbf1^120a^;f00391* eye discs. Interestingly, although the *UAS-Psc* construct causes ectopic cell death in wild-type eye discs, it is able to achieve a much higher level of cell death in *rbf1^120a^* mutant eye discs, suggesting that Psc may cooperate with *rbf1* mutations to promote cell death (Figure 3C). Nevertheless, these results suggest that the hypomorphic *rbf1* mutation does not necessarily cooperate with Psc overexpression to produce novel biological phenotypes, but rather represents a sensitized genetic background for Psc-induced phenotypes.

**Figure 4:**
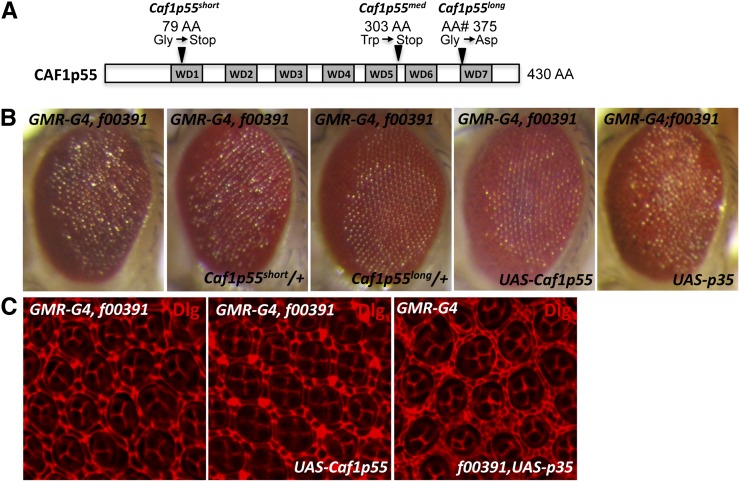
The Psc induced rough eye phenotype is suppressed by a specific *Caf1p55* mutant allele and *Caf1p55* overexpression. (A) A schematic representation of a PRC2 component, CAF1p55, containing seven WD40 domains, is shown. The *Caf1p55^short^* allele carries a point mutation that changes Gly to a stop codon at AA position 79, whereas the *Caf1p55^long^* alleles carries a point mutation that changes Gly to Asp at AA position 375 (Anderson *et al.* 2011). (B) To determine the effect of altering *Caf1p55* activity in Psc-induced rough eye phenotype, a single copy of either *Caf1p55^short^* mutant or *Caf1p55^long^* mutant chromosome was introduced in *GMR-Gal4;f00391* flies. In addition, wild-type CAF1p55 was coexpressed with Psc (*UAS-Caf1p55*). Note that both a single copy of the *Caf1p55^long^* mutation and CAF1p55 overexpression suppress the f00391-induced rough eye phenotype. Inhibiting cell death by coexpression of baculoviral protein p35 did not have a significant effect (*UAS-p35*). (C) Pupal eye discs overexpressing Psc alone or coexpressing CAF1p55 or p35 were immunostained for discs large (Dlg).

A recent study by (Anderson *et al.* 2011) reported the identification and characterization of *Caf1p55* mutant alleles. Interestingly, the loss-of-function phenotypes observed in *Caf1p55* mutant eyes are similar to those induced by Psc overexpression. Moreover, reduced *Caf1p55* activity leads to arista-to-tarsi transformation, a documented phenotype of Psc overexpression that we have observed with f00391 (Anderson *et al.* 2011; Sharp *et al.* 1994) (Supporting Information, Figure S1). To further investigate the relationship between *Psc* and *Caf1p55*, we took advantage of the fact that a stronger driver, *GMR-Gal4*, with f00391 (*GMR-Gal4;f00391*) can produce a dominantly modifiable rough eye phenotype in a wild-type background. Although *GMR-Gal4;f00391* failed to recapitulate the defect observed with ELAV staining, the same Senseless expression defect could be observed when Psc was overexpressed with *GMR-Gal4* (Figure S2A). Importantly, the adult eye phenotype of *GMR-Gal4;f00391* was suppressed by a single copy of an *Sce* mutant chromosome, which, like *Psc*, is a component of PRC1 (Figure S2B). This result demonstrated that the rough eye phenotype of *GMR-Gal4;f00391* could be used to identify factors that can dominantly modify the Psc-induced phenotype. Anderson *et al.* identified three *Caf1p55* alleles, *Caf1p55^short^*, *Caf1p55^med^*, and *Caf1p55^long^*. The *Caf1p55^short^* and *Caf1p55^med^* alleles have point mutations that introduce stop codons in the CAF1p55 coding region, whereas the *Caf1p55^long^* allele carries a point mutation that replaces the Gly^375^ with an Asp (Figure 4A). When we introduced a single copy of a chromosome carrying the *Caf1p55^short^* mutation, we did not observe any discernible effect on the *GMR-Gal4;f00391* rough eye phenotype (Figure 4B). However, introducing a single copy of the *Caf1p55^long^* mutant gene strongly suppressed the *GMR-Gal4;f00391* rough eye phenotype (Figure 4B). Interestingly, a single copy of the *Caf1p55^med^* mutation in *GMR-Gal4;f00391* flies resulted in adult eyes with varying degrees of roughness that ranged from a strong suppression to no effect on the rough eye phenotype (data not shown). Although the molecular nature of the mutation is unclear, one possibility for this observation is that the gene product of *Caf1p55^long^* retains some CAF1p55 function that is able to resist Psc overexpression. We then determined the effect of simply coexpressing wild-type CAF1p55 on the *GMR-Gal4;f00391* rough eye phenotype. As shown in Figure 4B, coexpression of CAF1p55 was able to strongly suppress the *GMR-Gal4;f00391*–induced rough eye phenotype. Coexpression of the baculoviral caspase inhibitor p35 was unable to suppress the eye phenotype, indicating that the ability of CAF1p55 to suppress the rough eye phenotype is specific (Figure 4B). Supporting this notion, analysis of pupal eye discs revealed that Caf1p55 suppressed the cone cell specification defect observed in *GMR-Gal4;f00391* flies, whereas baculoviral p35 did not (Figure 4C). These data suggest that the phenotype induced by Psc overexpression is highly sensitive to Caf1p55 activity and that Psc and CAF1p55 may have opposing effects when they are overexpressed.

**Figure 5:**
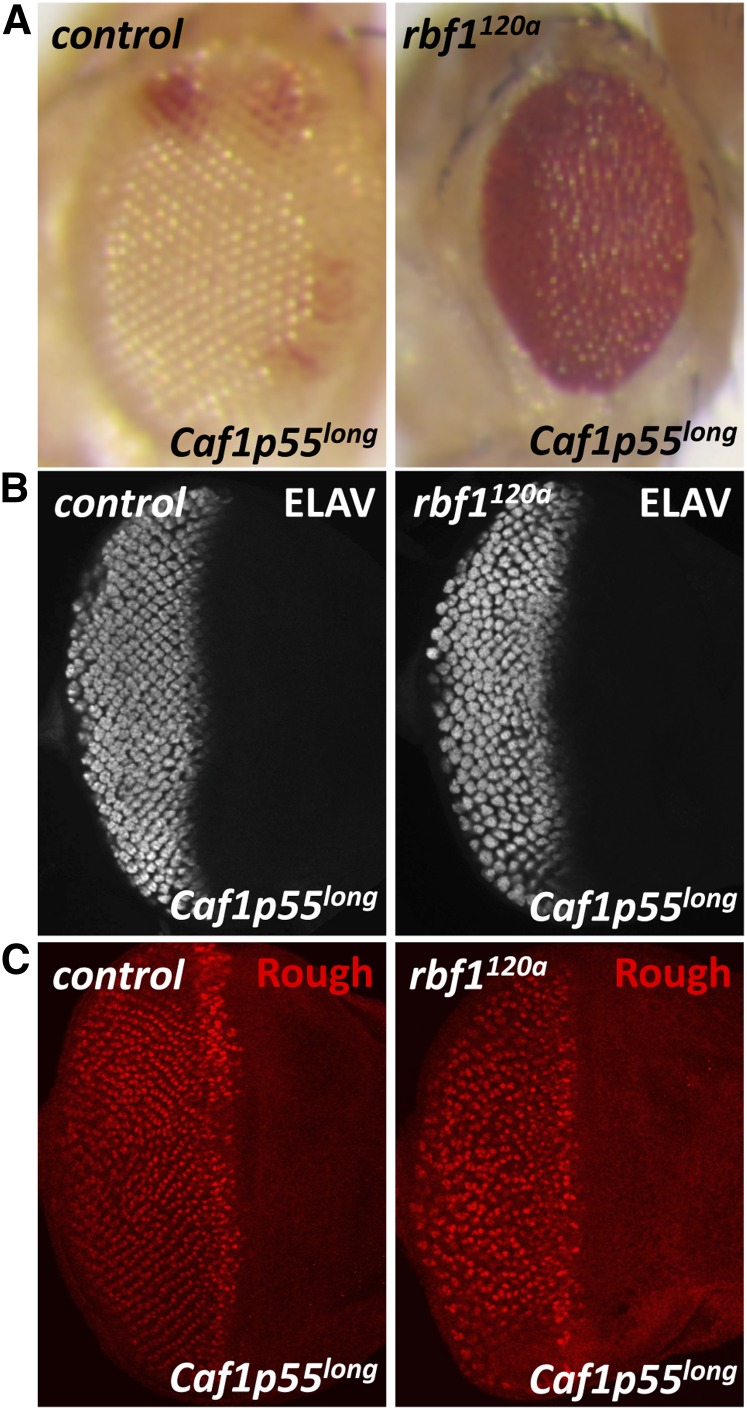
The *Caf1p55^long^* mutant allele genetically interacts with *rbf1* mutations. (A) Adult eyes composed primarily of *Caf1p55^long^* mutant cells in control and *rbf1^120a^* backgrounds were generated. *rbf1^120a^;Caf1p55^long^* double-mutant eyes are rougher and smaller compared with single-mutant *Caf1p55^long^* eyes. (B and C) Larval eye discs from these flies were immunostained for ELAV (B) or Rough (C) to monitor ommatidia. Note that photoreceptor organization is more severely disrupted in the *rbf1^120a;^Caf1p55^long^* double-mutant eye discs.

The genetic interaction between *Psc* and *Caf1p55* led us to test whether *rbf1* can also genetically interact with *Caf1p55*. Strong *Caf1p55* alleles, *Caf1p55^short^* or *Caf1p55^med^*, could not be used because Caf1p55 is normally required for eye development. Eyes homozygous for *Caf1p55^short^* or *Caf1p55^med^* mutations display strong developmental defects, including a massive amount of cell death (Anderson *et al.* 2011). Therefore, we took advantage of the hypomorphic *Caf1p55* allele, *Caf1p55^long^*, to generate eyes composed entirely of mutant cells in a wild-type or the *rbf1^120a^* mutant background. Strikingly, *rbf1^120a^;Caf1p55^long^* double-mutant adult eyes were smaller and rougher than *Caf1p55^long^* single-mutant eyes (Figure 5A). Third instar eye imaginal discs were immunostained for ELAV, to visualize all photoreceptors, and Rough, to look at photoreceptors R2 and R5. ELAV and Rough staining patterns showed that *rbf1^120a^;Caf1p55^long^* double mutants have greater disorganization of ommatidial clusters compared with single mutants (Figure 5, B and C). Interestingly, unlike *rbf1^120a^* eye discs overexpressing PSC, *rbf1^120a^;Caf1p55^long^* double-mutant eye discs did not show any effects on cell death, nor did they have defects in Senseless expression (data not shown). Because it remains unclear how the point mutation in *Caf1p55^long^* allele affects overall CAF1p55 function, we were unable to draw strong conclusions from the lack of discernible phenotypes. This result raises the possibility that the normal function of *Caf1p55* is compromised by *rbf1* mutations, providing a plausible explanation for the sensitivity of *rbf1* mutant cells to Psc overexpression.

**Figure 6:**
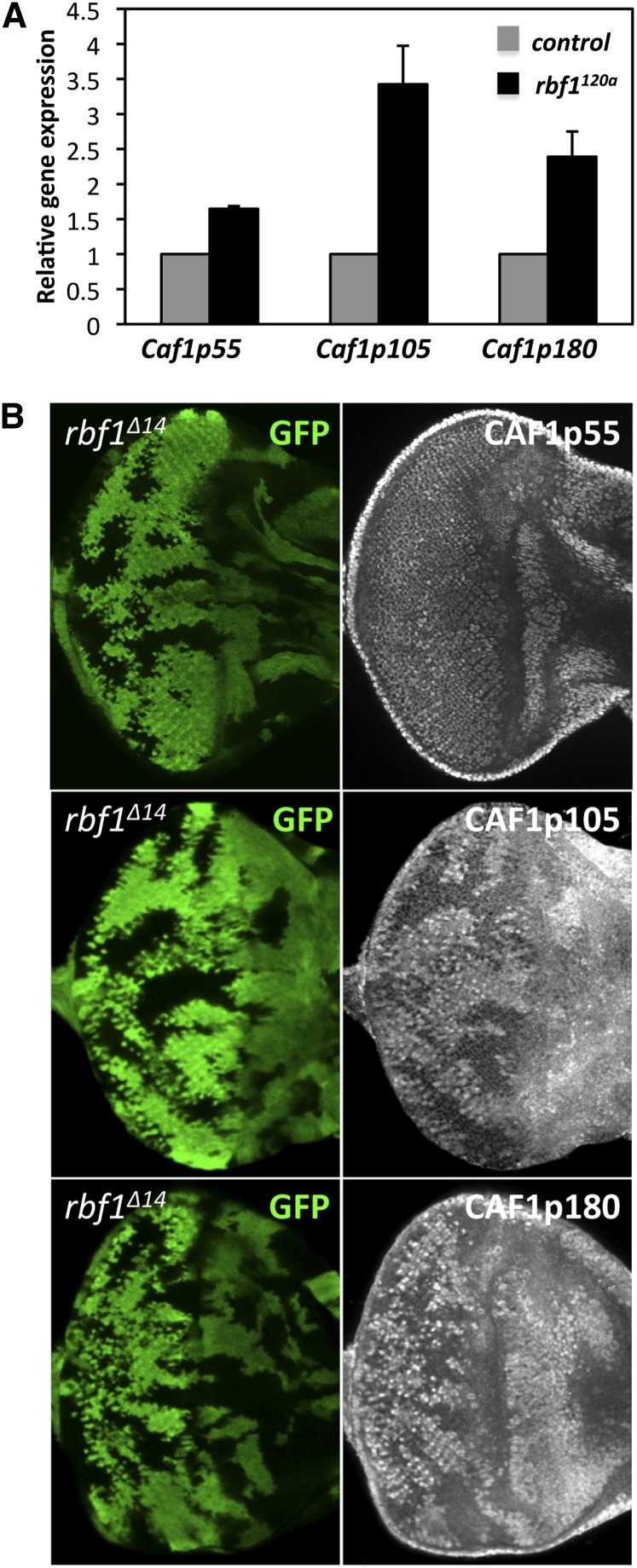
The expression levels of dCAF-1 components, *Caf1p105* and *Caf1p180*, are increased in *rbf1* mutant cells. (A) qRT-PCR showed elevated *Caf1p105* and *Caf1p180* expression in *rbf1^120a^* eye discs by 3.4-fold and 2.4-fold, respectively. *Caf1p55* transcripts were also increased 1.7-fold. The average fold change of three independent replicates compared with the control is represented. Error bars indicate the SD of three independent experiments. (B) Negatively marked (lack of GFP) *rbf1*-null clones were generated in eye discs using the *rbf1^Δ14^* allele. Immunostaining showed that the protein levels of dCAF1 components, CAF1p105 and CAF1p180, were increased in *rbf1*-null clones. Interestingly, CAF1p55 protein level was not significantly changed in *rbf1^Δ14^* clones.

The genetic interaction between *Caf1p55* and *rbf1* directed us to explore ways that CAF1p55 function might be affected by *rbf1* mutations. Aside from its function as a PRC2 component, CAF1p55 is part of many chromatin-associated complexes, including nucleosome remodeling factor, RBF-containing dREAM/MMB, and dCAF-1 (Kamakaka *et al.* 1996; Korenjak *et al.* 2004; Martinez-Balbas *et al.* 1998). One possible explanation for the genetic interaction between *Caf1p55* and *rbf1* is that the function of the dREAM complex, which includes both CAF1p55 and Rbf1 proteins, is compromised by *Caf1p55* and *rbf1* hypomorphic mutations. However, mutations in dE2F2 or mip130, components of the dREAM complex, are not known to induce severe developmental defects in the eye, indicating that the genetic interaction between *rbf1^120a^* and *Caf1p55^long^* cannot be explained solely by the inactivation of dREAM function (Beall *et al.* 2004; Frolov *et al.* 2001). Therefore, we asked if other factors compromise CAF1p55 function in *rbf1* mutant cells. When we analyzed microarray data from previous studies, we discovered that *Caf1p180* and *Caf1p105*, components of dCAF-1, are upregulated in *rbf1^120a^* eye discs (Nicolay *et al.* 2011). dCAF-1 is a H3/H4 histone chaperone complex composed of CAF1p180, CAF1p105, and CAF1p55 (Tyler *et al.* 2001). qRT-PCR confirmed that *Caf1p105* and *Caf1p180* transcript levels are increased 3.4-fold and 2.4-fold, respectively, in *rbf1* mutant eye discs compared with the wild-type (Figure 6A). The level of *Caf1p55* transcripts was also increased 1.7-fold. To determine if the protein expressions were also affected, we generated negatively marked *rbf1*-null clones using the *rbf1^Δ14^* allele and immunostained for dCAF-1 components. CAF1p105 and CAF1p180 protein levels were considerably increased in *rbf1*-null clones. Interestingly, the expression level of CAF1p55 protein was largely unchanged in *rbf1*-null clones (Figure 6B). Overall, only the protein expression of CAF1p55-interacting factors, CAF1p180 and CAF1p105, was upregulated by *rbf1* mutation.

**Figure 7:**
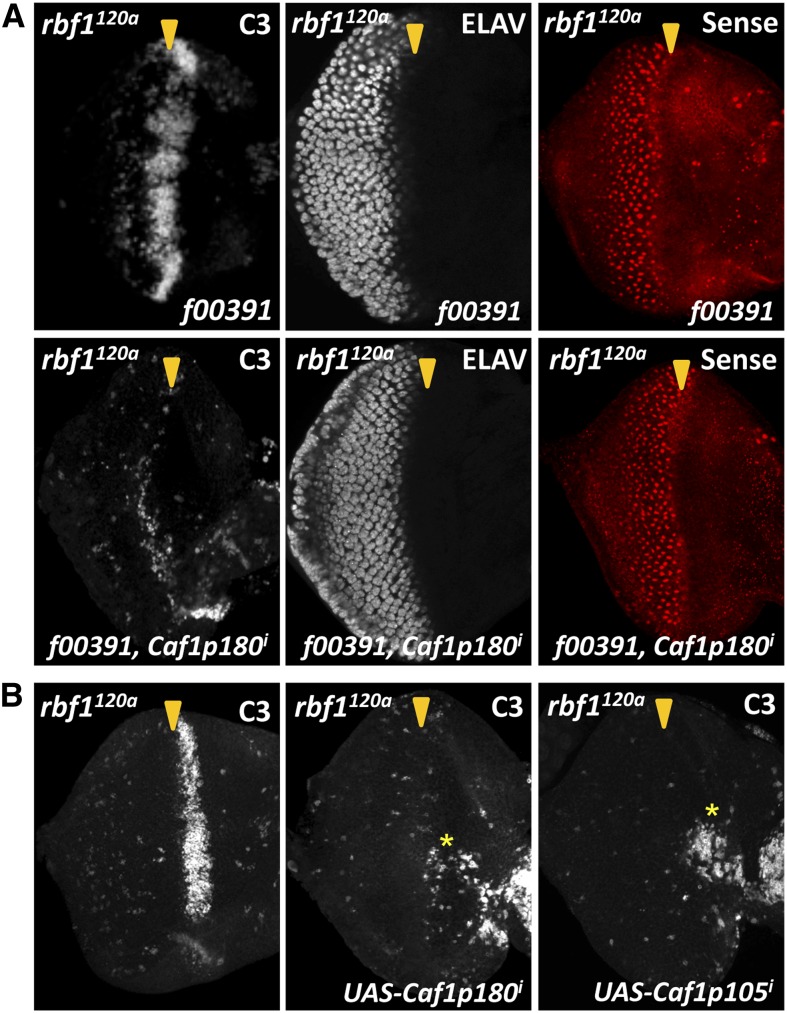
Knockdown of dCAF-1 components suppresses the cell death phenotype associated with *rbf1^120a^* eye discs but cannot suppress differentiation defects induced by Psc. (A) An RNAi construct for *Caf1p180* (*Caf1p180^i^*) was expressed with Psc (f00391) in *rbf1^120a^* eye discs and the pattern of cell death was visualized by anti-caspase-3 (C3) immuno staining. Note that the Psc-induced cell death was suppressed when *Caf1p180^i^* was coexpressed. Eye discs also were immunostained for ELAV to visualize ommatidial organization and for Senseless to determine R8 cell fate. *Caf1p180^i^* is unable to rescue the Psc-induced differentiation phenotypes. (B) RNAi constructs targeting *Caf1p180* or *Caf1p105* are expressed in the *rbf1^120a^* background and C3 marks dying cells. Note that knocking down *CAF1p105* or *CAF1p180* inhibits the cell death stripe normally observed in *rbf1^120a^* mutant eye discs. The depletion of *Caf1p180* or *Caf1p105* induces cell death in the anterior region of imaginal discs, where cells are actively dividing (asterisk).

We then asked whether the increased expression of dCAF-1 components in *rbf1* mutant eye discs is functionally significant. If the upregulation of CAF1p105 and CAF1p180 contributes to the sensitization of *rbf1* mutant cells to Psc overexpression, then decreasing the levels of *Caf1p105* and *Caf1p180* in *rbf1^120a^* eyes should suppress the Psc-induced phenotypes in *rbf1^120a^* mutant flies. Because *Caf1p180* is essential for *Drosophila* eye development (Song *et al.* 2007), we took advantage of a *Caf1p180* RNAi construct (*Caf1p180^i^*), which was able to substantially decrease CAF1p180 protein expression (Figure S3). Strikingly, coexpression of *Caf1p180^i^* strongly suppressed the cell death phenotype caused by f00391 in *rbf1^120a^* eyes, indicating that *Caf1p180* is required for sensitivity of *rbf1* mutant cells to Psc-induced cell death (Figure 7A). However, despite its effect on cell death, *Caf1p180^i^* did not suppress the f00391-induced ommatidial disorganization or the defect in Senseless expression, indicating that factors other than CAF1p180 are likely responsible for the sensitivity of *rbf1^120a^* eyes to Psc-induced differentiation defects (Figure 7A). We also noted that the stripe of cell death normally observed in *rbf1^120a^* eye discs was no longer present in *rbf1^120a^* eyes expressing both *Psc* and *Caf1p180* dsRNA (Figure 7A). This led us to test whether the increased expression of dCAF-1 components, *Caf1p105* and *Caf1p180*, is normally required for the developmentally regulated cell death in *rbf1* mutant eye discs. We used RNAi constructs targeting *Caf1p105* and *Caf1p180* to reduce their expression level in *rbf1^120a^* eye discs. We observed that RNAi against *Caf1p105* and *Caf1p180* induces ectopic cell death in the anterior region of the eye disc, where cells are asynchronously dividing, suggesting that dCAF-1 function is required for the survival of actively dividing cells (Figure 7B, asterisk). Importantly, the characteristic stripe of cell death present in *rbf1^120a^* eye discs disappeared when RNAi constructs targeting *Caf1p105* or *Caf1p180* were expressed (Figure 7B). These results indicate that deregulated expression of dCAF-1 components is required for the sensitivity of *rbf1* mutant cells to Psc-induced cell death, as well as for the developmentally regulated cell death associated with *rbf1* mutation.

## Discussion

In this study, we have described a genetic interaction between *rbf1* and a Polycomb group gene, *Psc*. In pursuing this genetic interaction, we discovered that Psc-induced phenotypes highly depend on the function of Caf1p55, a shared component of numerous chromatin-associated complexes including dCAF-1. Importantly, we provided evidence demonstrating that dCAF-1 activity is deregulated in *rbf1* mutant eyes, and that deregulated dCAF-1 activity is responsible for the sensitivity of *rbf1* mutant cells to Psc-induced cell death. Moreover, we also showed that deregulated dCAF-1 activity contributes to the cell death phenotype normally associated with *rbf1* mutations during *Drosophila* eye development.

In a serendipitous manner, we identified f00391 in a genetic screen that was designed to identify factors that induce eye phenotypes specifically in *rbf1* mutant flies. If the levels of Psc overexpression achieved by f00391 were comparable with that of the *UAS-Psc* transgene, then it is unclear whether f00391 would have been identified in the genetic screen. Nevertheless, Psc overexpression produced a plethora of developmental defects that ranged from ectopic cell death to failure to maintain Senseless expression in R8 cells. Our effort to determine the specific cell types that are affected by Psc overexpression did not yield any conclusive results. Immunostaining for specific photoreceptors and cone cells using Rough, Lozenge, Prospero, and Cut antibodies all showed patterning defects (data not shown), indicating that Psc overexpression interferes with broad developmental processes during *Drosophila* eye development. Given the scale of the biological processes in which PcG proteins participate, these findings were not unexpected (Janody *et al.* 2004).

Although the molecular mechanism underlying the genetic interaction between *Psc* and *Caf1p55* is unclear, it is probable that overexpressing Psc largely antagonizes CAF1p55 function. This idea is supported by the observation that Psc overexpression induces arista-to-tarsi transformation, a phenotype observed when CAF1p55 activity is compromised, and by the discovery that the rough eye phenotype induced by *GMR-Gal4;f00391* is strongly suppressed by coexpression of CAF1p55 (Figures S1 and Figure 4B). We also discovered that the *GMR-Gal4;f00391* rough eye phenotype could be dominantly enhanced by mutations in the genes encoding proteins that physically associate with CAF1p55, including PRC2 components (Figure S4). However, without delving into the exact molecular mechanism, our genetic study does not address whether all CAF1p55-associated complexes are equally affected by Psc overexpression. Moreover, the Senseless expression defect caused by Psc overexpression was not observed in the *Caf1p55* mutant study by Anderson *et al.* indicating that Psc overexpression may have differential effects on CAF1p55-associated complexes or CAF1p55-independent effects. Interestingly, the *Caf1p55^long^* allele was able to dominantly suppress the *GMR-Gal4;f00391* rough eye phenotype, whereas a stronger *Caf1p55* allele, *Caf1p55^short^*, could not (Figure 4B). We speculate that the mutant CAF1p55 protein expressed by the *Caf1p55^long^* allele retains some CAF1p55 function and is able to resist the effect of Psc overexpression. It will be interesting to investigate the molecular nature of the *Caf1p55^long^* mutation, which will likely provide clues regarding how Psc overexpresion affects CAF1p55 activity. It is important to note that the expression of BMI-1, the mammalian homolog of Psc, is upregulated in a subset of cancers (Guo *et al.* 2011; Qin *et al.* 2009). Given the number of epigenetic regulators that contain CAF1p55, our findings raise the possibility that the oncogenic activation of BMI-1 may lead to a broad range of epigenetic changes that are not restricted to the function of BMI-1 as a PcG protein.

We demonstrated that the increased expression level of CAF1p180 and CAF1p105 contributes to the genetic interaction between *Psc* and *rbf1*, as well as to the developmentally regulated cell death normally observed in *rbf1* mutant cells (Figure 7). One interesting speculation from our study is that *rbf1* mutation alters the stoichiometry of CAF1p55-associated complexes by increasing CAF1p180 and CAF1p105 levels without affecting CAF1p55 level. This would sensitize *rbf1* mutant cells to Psc overexpression, which can also influence CAF1p55 function. In an attempt to test this model, we overexpressed CAF1p55 in an *rbf1* mutant background. However, unlike *Caf1p180* or *Caf1p105* knockdown, overexpressing CAF1p55 did not inhibit the cell death stripe in *rbf1* eye discs, indicating that CAF1p55 level is not limiting in *rbf1* mutant eye discs (Figure S5A). Importantly, Caf1p55 overexpression did suppress the Psc-induced cell death phenotype in an *rbf1* mutant background, again supporting the notion that Psc and Caf1p55 can antagonize each other when they are overexpressed (Figure S5B). An alternative model to explain the effect of *Caf1p180* and *Caf1p105* depletion on the *rbf1* mutant cell death stripe in eye discs is that deregulated dCAF-1 activity may function independently of other CAF1p55-associated complexes. dCAF-1 has been shown to be involved in transcriptional regulation such as in heterochromatin formation (Huang *et al.* 2010). In this instance, CAF1p55 inactivation should have a similar effect on the stripe of cell death as *Caf1p180* or *Caf1p105* depletion. We tested this model by expressing an RNAi construct targeting *Caf1p55* in *rbf1^120a^* eye discs. Depletion of Caf1p55 in *rbf1* mutant eyes did inhibit the stripe of cell death, supporting the idea that deregulated dCAF-1 activity itself contributes to this phenotype (Figure S5A). However, because Caf1p55 depletion itself induces a strong cell death phenotype in both control and *rbf1* mutant eyes, we could not make a strong conclusion from this result. Whether deregulated dCAF-1 activity can affect the function of other CAF1p55-associated complexes, our genetic data clearly show that increased expression of CAF1p180 and CAF1p105 is necessary for the ability of Psc to promote cell death in *rbf1* mutant cells. Interestingly, overexpression of CAF1p180 has been shown to induce cell death during *Drosophila* eye development (Song *et al.* 2007). However, the exact molecular mechanism by which CAF1p180 promotes cell death remains unclear. It is also important to remember that factors other than CAF1p180 and CAF1p105 likely contribute to the genetic interaction between *Psc* and *rbf1* because the differentiation defects were not suppressed by *Caf1p180* or *Caf1p105* RNAi (Figure 7).

Previous studies have focused mainly on the direct targets of E2F transcription factors to understand the transcriptional consequence of pRB deficiency. Our study provides evidence to suggest that pRB deficiency deregulates CAF1p55 and possibly alters the activity of CAF1p55-associated protein complexes. Interestingly, a recent study showed that retinoblastoma genomes are more stable than previously thought, whereas a large number of epigenetic changes are commonly found in these tumors (Zhang *et al.* 2012). Clearly, the relationship between pRB status and epigenetic alterations warrants further investigation.

## Supplementary Material

Supporting Information
