# Supplementary material for: The Components of Drosophila Histone Chaperone dCAF-1 Are Required for the Cell Death Phenotype Associated with rbf1 Mutation
Source: G3 (Bethesda). 2013 Oct 1;3(10):1639–47. doi: 10.1534/g3.113.007419 (PMC3789789; doi:10.1534/g3.113.007419)
Supplement: Supporting Information [file supp_3_10_1639__index.html]

The Components of Drosophila Histone Chaperone dCAF-1 Are Required for the Cell Death Phenotype Associated with rbf1 Mutation — Supporting Information 

# The Components of *Drosophila* Histone Chaperone dCAF-1 Are Required for the Cell Death Phenotype Associated with *rbf1* Mutation

## Supporting Information for Collins and Moon, 2013

**Files in this Data Supplement:**

- Supporting Information - Figures S1-S5 (PDF, 825 KB)
- Figure S1 - Psc overexpression induces arista-to-tarsi transformation. (PDF, 331 KB)
- Figure S2 - Psc expression using a GMR-Gal4 driver induces an adult eye phenotype in a wild-type background. (PDF, 398 KB)
- Figure S3 - Expression of CAF1p180 is reduced by an RNAi construct. (PDF, 338 KB)
- Figure S4 - *GMR-G4,f00391* adult eye phenotype is dominantly enhanced by CAF1p55-interacting components. (PDF, 383 KB)
- Figure S5 - Effect of CAF1p55 overexpression and depletion on the pattern of cell death in *rbf1* mutant eye discs (PDF, 439 KB)
