# Supplementary material for: The Components of Drosophila Histone Chaperone dCAF-1 Are Required for the Cell Death Phenotype Associated with rbf1 Mutation
Source: G3 (Bethesda). 2013 Oct 1;3(10):1639–47. doi: 10.1534/g3.113.007419 (PMC3789789; doi:10.1534/g3.113.007419)
Supplement: Supporting Information [file supp_g3.113.007419_007419SI.pdf]

G3·Genes | Genomes | Genetics

The Components of *Drosophila* Histone Chaperone dCAF-1 are Required for the Cell Death Phenotype Associated with *rbf1* mutation

Heather Collins and Nam-Sung Moon

Department of Biology, Developmental Biology Research Initiative, McGill University, Montreal, Quebec H3A 1B1, Canada.

DOI: 10.1534/g3.113.007419

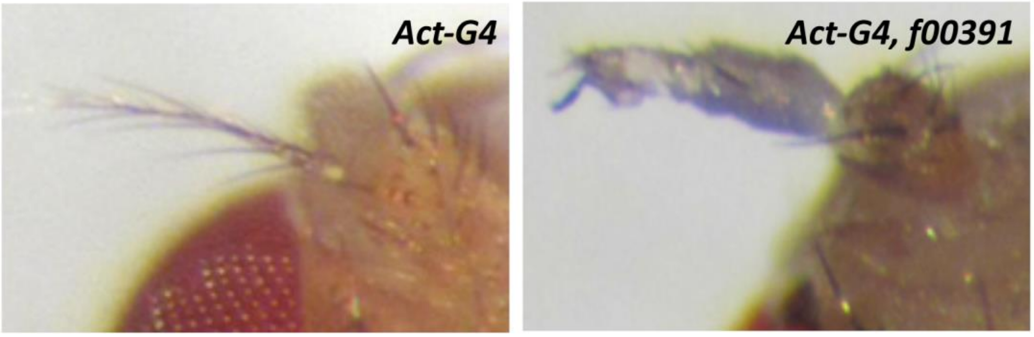

Figure S1 Psc overexpression induces arista-to-tarsi transformation. (A) Anderson et al., 2011 demonstrated that the arista of *Drosophila* antenna transforms to tarsi of the leg when the activity of CAF1p55 is compromised. Overexpression of Psc from f00391 in an eye-antennal-specific manner also induces arista-to-tarsi transformation, mimicking the developmental phenotype associated with compromised CAF1p55 function.

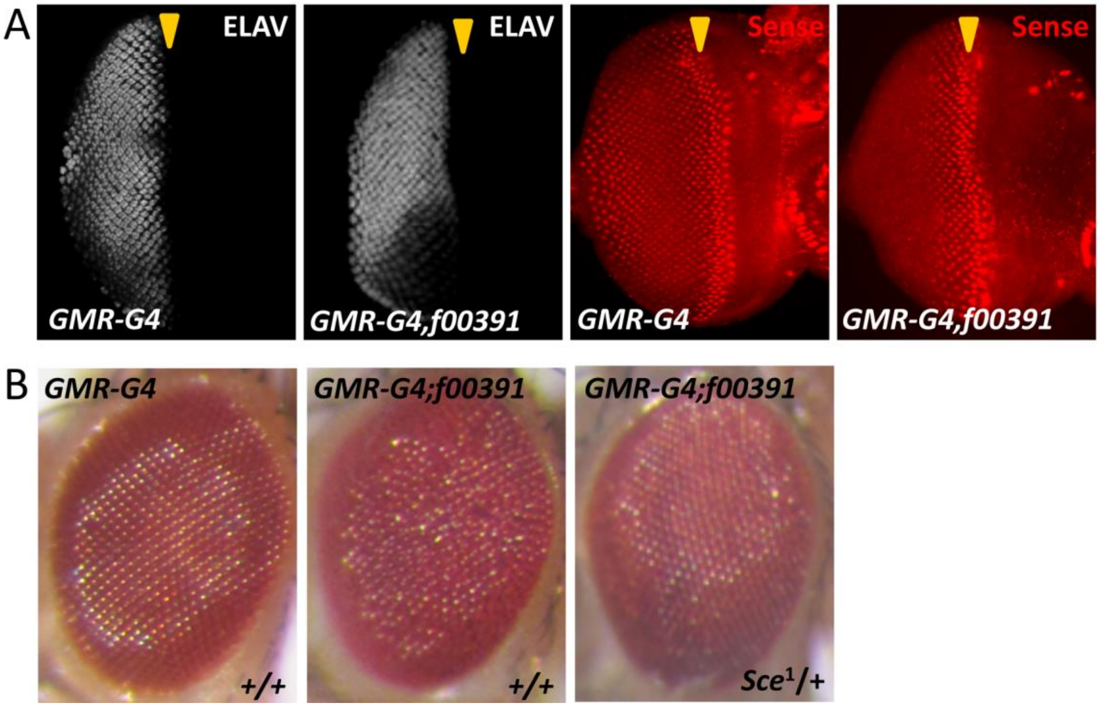

Figure S2 Psc expression using a GMR-Gal4 driver induces an adult eye phenotype in a wild-type background. (A) GMR-Gal4 was used to drive expression of Psc from f00391 (*GMR-G4,f00391*). Eye imaginal discs were stained for ELAV and Senseless to visualize photoreceptor differentiation. Eye discs with the GMR-Gal4 driver alone were used as a control (*GMR-G4*). Note that although the ELAV pattern appears normal, the Senseless expression is lost in ommatidia located at the posterior region of the eye disc. (B) The adult eye of *GMR-G4* and *GMR-G4,f00391* flies are shown. Note that the stereotypic pattern of the adult eye is disrupted in *GMR-G4,f00391* flies unlike *Act-G4;f00391* flies (Figure 1A). Importantly, introducing a single copy of the chromosome carrying mutations of a PRC1 component ($Sce^1/+$) considerably suppresses the adult eye phenotype.

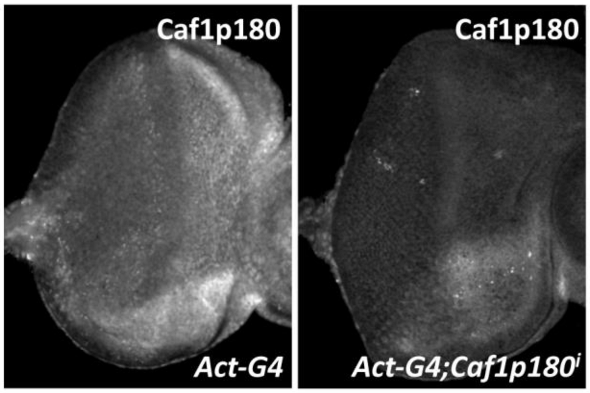

Figure S3 Expression of CAF1p180 is reduced by an RNAi construct. An RNAi construct targeting *Caf1p180* was expressed in eye imaginal discs. Eye discs were immunostained for CAF1p180 and a substantial decrease in protein expression could be observed, demonstrating that the RNAi construct is functioning as expected.

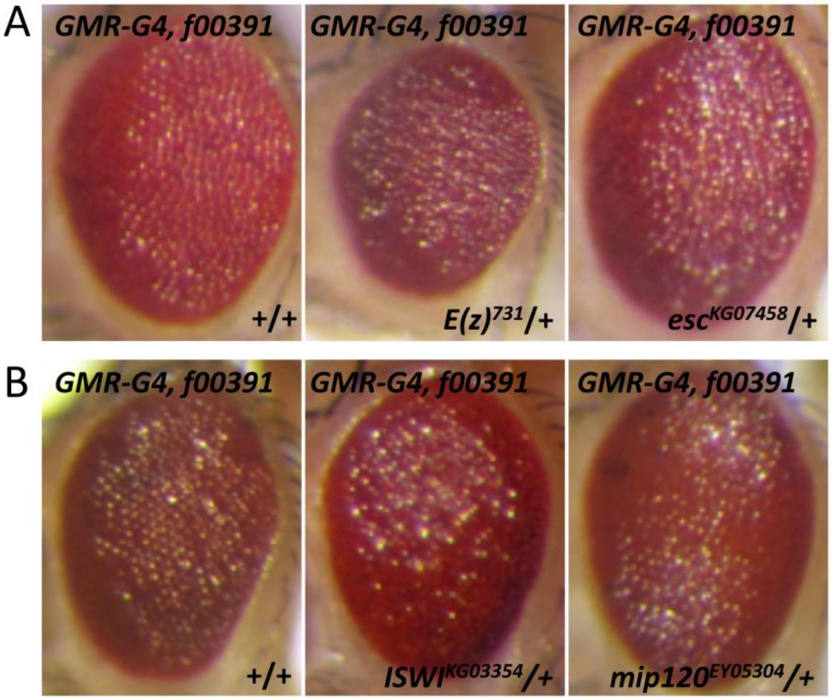

Figure S4 *GMR-G4,f00391* adult eye phenotype is dominantly enhanced by CAF1p55-interacting components. (A) Mutations of PRC2 components, *E(Z)* and *esc*, dominantly enhanced the Psc-induced eye phenotype. These results support the notion that Psc overexpression antagonizes the function of Caf1p55, which is a shared component of numerous epigenetic regulators. (B) Introducing a single mutant copy of the genes encoding Caf1p55-interacting proteins, ISWI (a component of NURF) and mip120 (a component of dREAM), enhanced the Psc-induced eye phenotype.

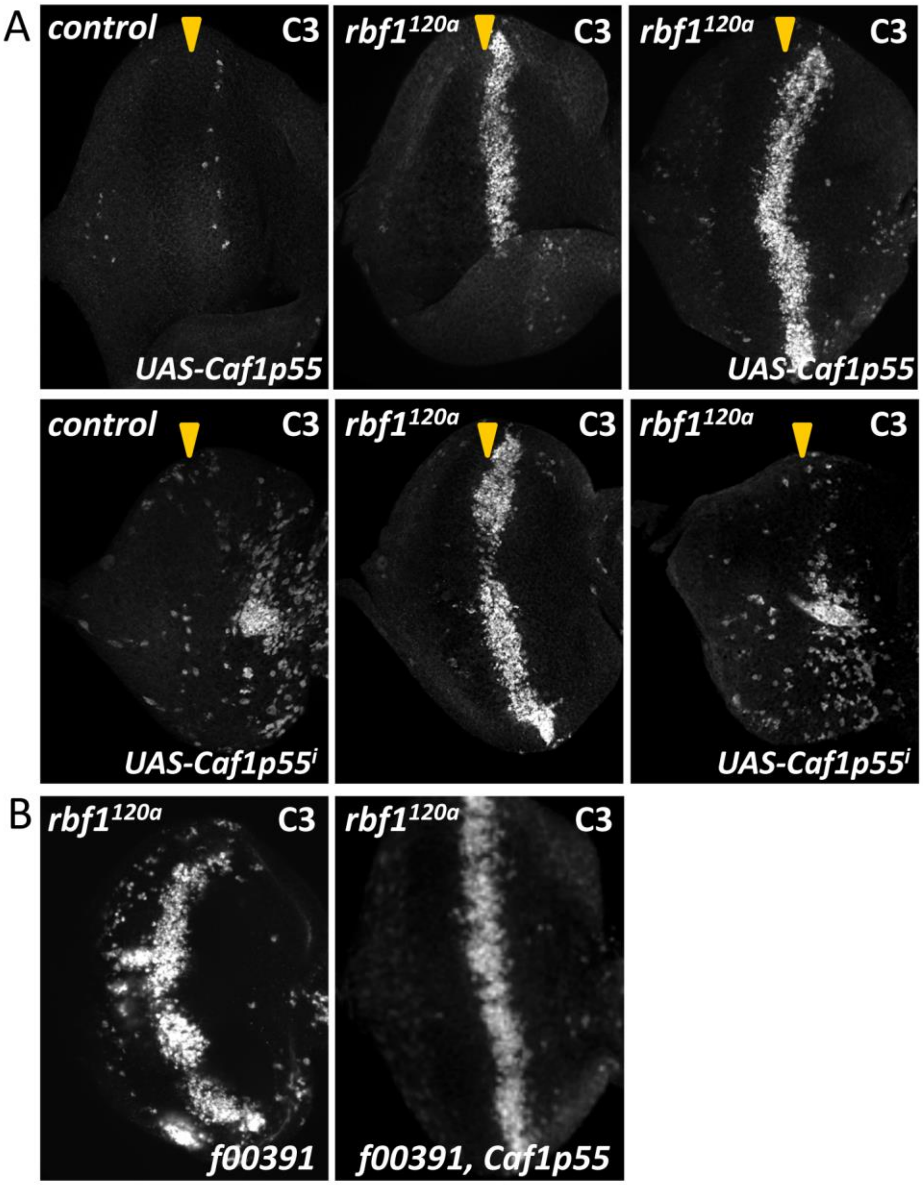

Figure S5 Effect of CAF1p55 overexpression and depletion on the pattern of cell death in *rbf1* mutant eye discs. (A) UAS-Caf1p55 or an RNAi construct targeting *Caf1p55 (Caf1p55i)* was used to either overexpress or deplete CAF1p55 in control and *rbf1*^{120a} eye discs. Eye discs were immunostained for C3 to monitor apoptotic cells. (B) Psc was expressed alone (f00391) or co-expressed with Caf1p55 (f00391, Caf1p55) in *rbf1*^{120a} eye discs. Anti-cleaved C3 was used to monitor dying cells. Note that the Psc-induced ectopic cell death phenotype is suppressed by CAF1p55 co-expression, but the stripe of cell death in *rbf1* mutant eye discs is still present.
