# Supplementary material for: The Components of Drosophila Histone Chaperone dCAF-1 Are Required for the Cell Death Phenotype Associated with rbf1 Mutation
Source: G3 (Bethesda). 2013 Oct 1;3(10):1639–47. doi: 10.1534/g3.113.007419 (PMC3789789; doi:10.1534/g3.113.007419)
Supplement: Supporting Information [file supp_g3.113.007419_FigureS1.pdf]

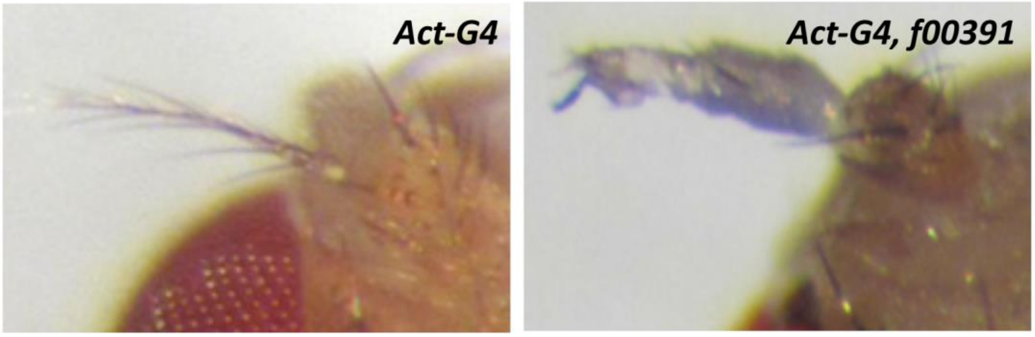

Figure S1 Psc overexpression induces arista-to-tarsi transformation. (A) Anderson et al., 2011 demonstrated that the arista of *Drosophila* antenna transforms to tarsi of the leg when the activity of CAF1p55 is compromised. Overexpression of Psc from f00391 in an eye-antennal-specific manner also induces arista-to-tarsi transformation, mimicking the developmental phenotype associated with compromised CAF1p55 function.
