# Supplementary material for: The Components of Drosophila Histone Chaperone dCAF-1 Are Required for the Cell Death Phenotype Associated with rbf1 Mutation
Source: G3 (Bethesda). 2013 Oct 1;3(10):1639–47. doi: 10.1534/g3.113.007419 (PMC3789789; doi:10.1534/g3.113.007419)
Supplement: Supporting Information [file supp_g3.113.007419_FigureS2.pdf]

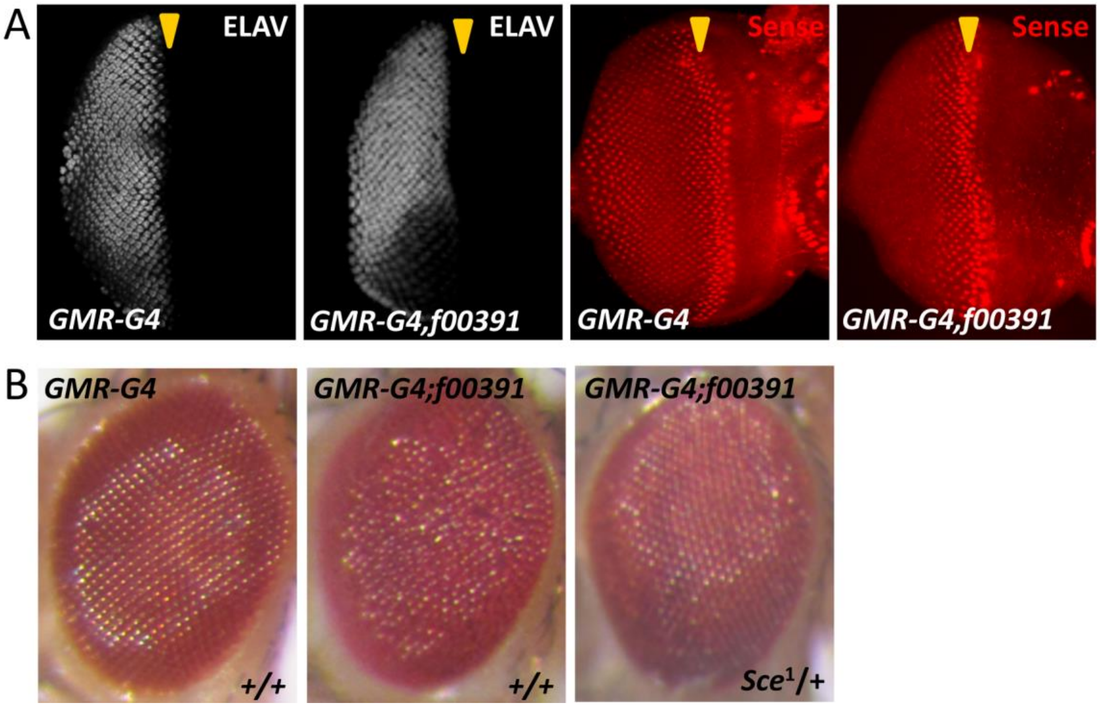

Figure S2 Psc expression using a GMR-Gal4 driver induces an adult eye phenotype in a wild-type background. (A) GMR-Gal4 was used to drive expression of Psc from f00391 (*GMR-G4,f00391*). Eye imaginal discs were stained for ELAV and Senseless to visualize photoreceptor differentiation. Eye discs with the GMR-Gal4 driver alone were used as a control (*GMR-G4*). Note that although the ELAV pattern appears normal, the Senseless expression is lost in ommatidia located at the posterior region of the eye disc. (B) The adult eye of *GMR-G4* and *GMR-G4,f00391* flies are shown. Note that the stereotypic pattern of the adult eye is disrupted in *GMR-G4,f00391* flies unlike *Act-G4;f00391* flies (Figure 1A). Importantly, introducing a single copy of the chromosome carrying mutations of a PRC1 component (*Sce*^{1}*/+*) considerably suppresses the adult eye phenotype.
