# Supplementary material for: The Components of Drosophila Histone Chaperone dCAF-1 Are Required for the Cell Death Phenotype Associated with rbf1 Mutation
Source: G3 (Bethesda). 2013 Oct 1;3(10):1639–47. doi: 10.1534/g3.113.007419 (PMC3789789; doi:10.1534/g3.113.007419)
Supplement: Supporting Information [file supp_g3.113.007419_FigureS3.pdf]

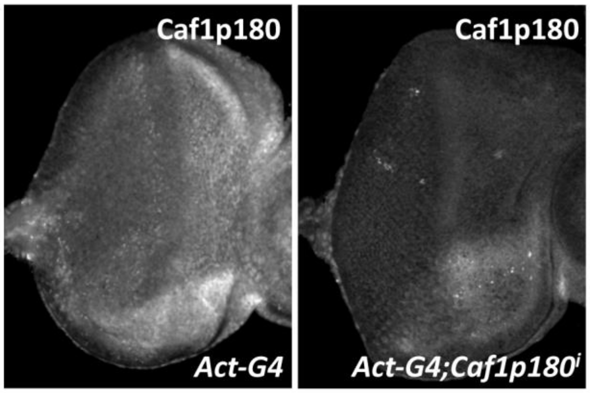

Figure S3 Expression of CAF1p180 is reduced by an RNAi construct. An RNAi construct targeting *Caf1p180* was expressed in eye imaginal discs. Eye discs were immunostained for CAF1p180 and a substantial decrease in protein expression could be observed, demonstrating that the RNAi construct is functioning as expected.
