# Supplementary material for: The Components of Drosophila Histone Chaperone dCAF-1 Are Required for the Cell Death Phenotype Associated with rbf1 Mutation
Source: G3 (Bethesda). 2013 Oct 1;3(10):1639–47. doi: 10.1534/g3.113.007419 (PMC3789789; doi:10.1534/g3.113.007419)
Supplement: Supporting Information [file supp_g3.113.007419_FigureS4.pdf]

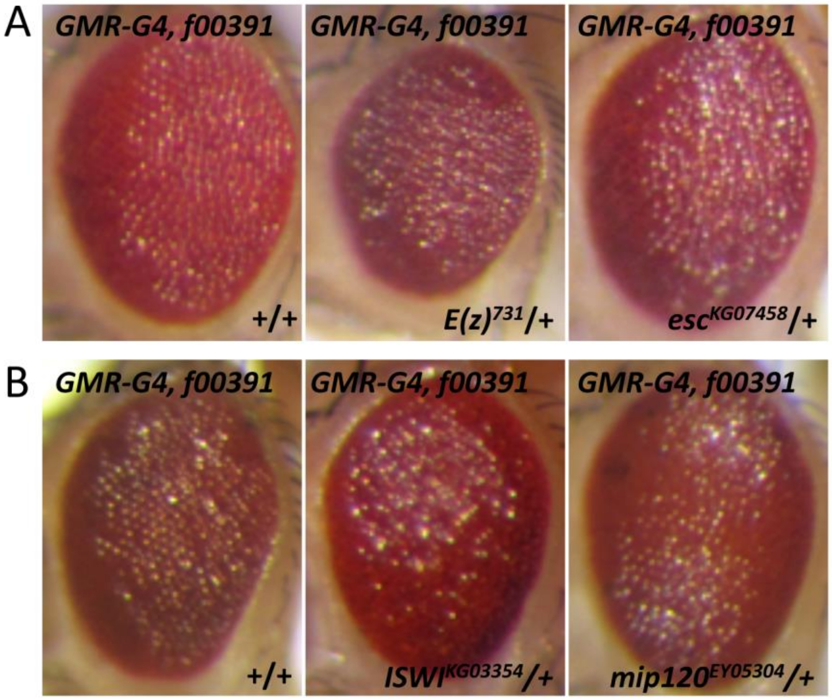

Figure S4 *GMR-G4,f00391* adult eye phenotype is dominantly enhanced by CAF1p55-interacting components. (A) Mutations of PRC2 components, *E(Z)* and *esc*, dominantly enhanced the Psc-induced eye phenotype. These results support the notion that Psc overexpression antagonizes the function of Caf1p55, which is a shared component of numerous epigenetic regulators. (B) Introducing a single mutant copy of the genes encoding Caf1p55-interacting proteins, ISWI (a component of NURF) and mip120 (a component of dREAM), enhanced the Psc-induced eye phenotype.
