# Supplementary material for: The Components of Drosophila Histone Chaperone dCAF-1 Are Required for the Cell Death Phenotype Associated with rbf1 Mutation
Source: G3 (Bethesda). 2013 Oct 1;3(10):1639–47. doi: 10.1534/g3.113.007419 (PMC3789789; doi:10.1534/g3.113.007419)
Supplement: Supporting Information [file supp_g3.113.007419_FigureS5.pdf]

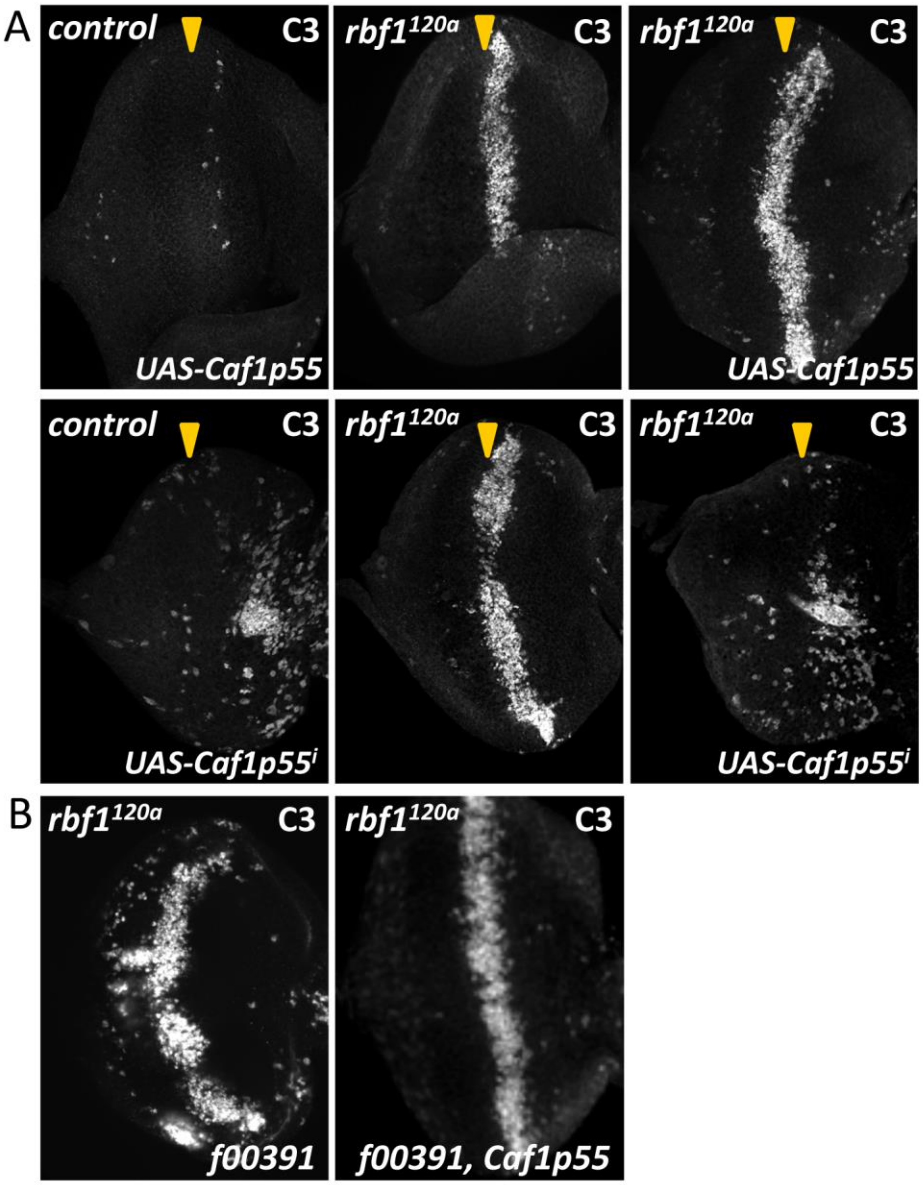

Figure S5 Effect of CAF1p55 overexpression and depletion on the pattern of cell death in *rbf1* mutant eye discs. (A) UAS-Caf1p55 or an RNAi construct targeting *Caf1p55 (Caf1p55i)* was used to either overexpress or deplete CAF1p55 in control and *rbf1*^{120a} eye discs. Eye discs were immunostained for C3 to monitor apoptotic cells. (B) Psc was expressed alone (f00391) or co-expressed with Caf1p55 (f00391, Caf1p55) in *rbf1*^{120a} eye discs. Anti-cleaved C3 was used to monitor dying cells. Note that the Psc-induced ectopic cell death phenotype is suppressed by CAF1p55 co-expression, but the stripe of cell death in *rbf1* mutant eye discs is still present.
